# The importance of the taste preferences and sensitivity of mothers and their children in the aspect of excessive body weight of children

**DOI:** 10.3389/fendo.2022.1031884

**Published:** 2022-11-16

**Authors:** Grzegorz Sobek, Mariusz Dąbrowski

**Affiliations:** ^1^ Institute of Health Sciences, College of Medical Sciences, University of Rzeszow, Rzeszów, Poland; ^2^ Institute of Medical Sciences, College of Medical Sciences, University of Rzeszow, Rzeszów, Poland

**Keywords:** children, mothers, taste preferences, taste perception, overweight and obesity

## Abstract

**Introduction:**

Food selection among adults and mostly children depends mainly on the taste of a dish. Poor taste sensitivity as well as strong preferences for sweet and fat taste may be the factors predisposing children to become overweight and/or develop obesity. Family environment, including mothers’ eating habits and preferences, may affect children’s taste perception and preferences. The aim of the study was to assess taste perception and preferences in children and their mothers in relation to their weight status.

**Methods:**

Sensory tests were carried out using puddings with different sugar and fat content. In all study participants anthropometric measurements (weight and height with BMI calculation) were performed.

**Results:**

The study results did not reveal any differences in the taste sensitivity of overweight/obese and normative body weight children. Similarity was found in the perception of different levels of sweet/fat flavors among children and parents. Overweight/obese children were two times more likely to choose a very fat and very sweet taste compared to normal weight children. The results showed that children prefer a sweet taste more often than their mothers. Mothers’ fat taste preferences were important - the fatter the taste they selected, the greater the percentage of children with obesity.

**Discussion:**

Mothers’ taste sensitivity may affect children’s perception of the quality (intensity) of flavors. Normal-weight children chose a low fat and low sweet taste more frequently than those with excess of body weight. The role of parents in shaping taste preferences is of utmost importance and should be based on limiting the consumption of products rich in sugar and/or fat.

## Introduction

Human food intake is regulated by a complex physiological mechanism associated not only with the feeling of hunger and satiety, but also with the perception of sensory stimuli. Food assessment is largely based on sensory impressions such as look, smell and finally taste. All basic flavors, i.e. sweet, bitter, salty, sour, umami, as well as fat play the role of food quality markers (1–3). Studies clearly show that sensory performance of the taste recognition apparatus is an individual feature that can affect the perception of food products and daily choices of food consumed (4, 5). The reasons for different sensitivity are complex and are the result of numerous factors including genetic, physiological and environmental ones. Factors that affect sensory perception can also be associated with age, sleep, body mass index, anxiety level and neurotransmitters, hormonal factors, and habitual diet (6). It is hypothesized that those less able to detect fatty acids (hyposensitive) appear to have, a higher body mass index (BMI) (7, 8). There are no clear data to confirm this, but several testable theory may be proposed. A frequently investigated hypothesis is that overweight and obese individuals are less sensitive to palatable fatty texture (mouthfeel) and therefore need a greater concentration to detect fatty mouthfeel (9).

Given that food preferences are an extremely important factor influencing diet quality, understanding how they change and how they can be modified can help to promote a healthy diet for both children and adults. Taste preferences can therefore be defined as making a food selection based on one’s own subjective, hedonic (“pleasant”, “unpleasant”) perceptions. In practice, due to the strong relationship between taste preferences and food selection, the term food preferences is often used, which means that we like some food products more or less, and we have an aversion towards others (10). Current research results indicate that children perceive some flavors differently than adults. Preferences of the youngest for higher levels of salty and sweet taste can be justified by evolutionary factors (11). The first reports describing the relationship between taste preferences, food consumption and obesity suggested that a greater intensity of taste contributes to the improvement of the palatability of food products, and thus to over-consumption and possible obesity (12). Lack of conclusive evidence in a few subsequent studies is associated with the heterogeneity of methods for measuring taste preferences and food consumption. Doubts also relate to the use of flavors in the form of laboratory samples in sensory tests due to their insufficient relation to consumption in real conditions (13).

Parents play a significant role in shaping the taste preferences of their children and adolescents. Their participation in developing sensory experiences is not limited to “setting an example,” but can have a much broader context. Parents can consciously or unconsciously control the availability of food products, and thus affect the exposure of different flavors (14).

Several reports are available in the literature analyzing the impact of children’s taste perception preferences in the context of overweight and/or obesity as well as adult taste preferences and perceptions separately (15–17). So far, there are no reports in the literature comparing the taste preferences and taste sensitivity of children and their parents with the state of body weight.

## Materials and methods

### Subject

The study is a preliminary assessment of the relationship between the taste perception and taste preferences of mothers and their children in relation to the BMI of mothers and children. 239 children aged 8-15 years were recruited for the study from two schools selected *via* an randomized algorithm, one in an urban (Stanisław Wyspiański Secondary School Complex No. 3. in Rzeszów, Poland) and one in a rural area (Complex of Schools in Kosina, Poland). The study group consisted of 75 children with overweight or obesity aged from 8 to 15 years. The control group consisted of 75 children with normal weight aged from 8 to 15 years, strictly matched to the study group regarding sex and age (the nearest date of birth for each comparator from the study group, in 1:1 ratio). The remaining 89 children not strictly matching to the study group regarding sex and age were excluded. All mothers (n=150) of the pediatric subjects wereincluded in the study. Mothers were chosen because of their impact on the development of childhood eating habits. 69 of them were overweight and/or obesity (BMI ≥25 kg/m^2^) (18). The inclusion criteria for children were age 8–15, the attendance of one of two selected schools, and parents’ acceptance to participate in the study. The requirement for the child’s participation in the study was the simultaneous participation of his/her mother. The exclusion criteria for children included suffering from chronic diseases affecting body weight, being underweight (<5th percentile), inability to consume food samples used in the study, implanted pacemaker and pregnancy (contraindications for bioimpedance testing). The children participating in the study were a representative sample of the population. The inclusion and exclusion criteria for mothers were the same, except for the age and body weight. During the parents meeting with teachers at schools, the main goals of the study were presented. Attending mothers were asked to participate in the study by themselves and to permit their children to participate. It was noted that the research is voluntary and has only a scientific purpose. The study was conducted after obtaining written consent from the participating children’s parents and the children themselves. All participants and parents were fully informed in writing and verbally about the nature of the study.

### Assessments of preferences and sensitivity

The study was conducted according to the protocol used to assess sweet and fat taste perception in the “I Family” study (funded by the EC FP7 project No 266044) using puddings with different sugar content and with different fat content (SOP - Carrying out a taste intensity test with children, adolescents and their parents to assess sweet and fat taste perception of different puddings) (19). The subjects task was to assess the intensity of three pudding samples with different fat content and three pudding samples with different sugar content. The evaluators were to rank three samples according to increasing fat content and another three according to increasing sugar content. In addition, the evaluators had to indicate which of the samples with different fat content and samples with different sugar content they like best. Between each test sequence the participants rinsed their mouth with demineralised water to avoid adaptation and took a two to three minutes break. The test continued with the second block (taste) following the same procedure. The pudding samples were presented under a red light in order to mask colour differences. Cold whipped vanilla pudding (RUF Schlemmer Crème, vanilla flavour) was a carrier of taste with different concentrations of sugar and fat. Base samples for both flavors were identical and contained 14.5% sugar and 3.1% fat. Modified samples to examine the perception of fat taste contained sugar base amount and increased fat concentration, i.e. 6.8% and 14.1%. In case of samples for the assessment of sweet taste, the base amount of fat was retained, and the amount of sugar was modified to 24.1% and 36.2%. All participants were given a template on which they had to complete a scale of taste perception intensity rating for each taste and concentration. The scale consisted of 3 intensity values (from 1 to 3), 1 meaning “low fat/sweet” and 3 “high fat/sweet”.

The interpretation of the results consisted in assessing the accuracy of ordering the intensity of the fat/sweet taste from the least fat/sweet to the high fat/sweet. In the case when all (three) samples were correctly ranked by participant, the test result was marked as maximum accuracy. When one or two errors were made, the test result was classified as average accuracy, while in the case when all the samples were incorrectly ranked, the test result was rated as lack of accuracy. For the fat taste the term “creamy” was used to avoid negative associations with the word fat.

### Anthropometric measurements of the studied group

Height measurements were made three times with the SECA 213 portable stadiometer, with an accuracy of 5 mm, in a standing position, upright, without footwear. The average figure of the three measurements was used in the analyzes. Body weight was assessed with an accuracy of 0.1 kg using a body composition analyzer (BC-420, Tanita, Tokyo, Japan). According to the instructions for the Tanita BC 420 device for accurate measurements, the machine was positioned as horizontally as possible. Participants stood on the platform barefoot, upright, on straight legs and made sure that the front of the feet touched the front electrodes and rear parts of the rear electrodes. The height and weight of all participants (children, mothers) were measured in fasting status wearing underwear. Body mass index (BMI) was calculated as weight (kg)/height (m)^2^. Based on BMI values, the BMI percentile of individual childrens was calculated. BMI percentile charts specific for age, sex, and body height were used. Percentile charts which were developed within the framework of the Polish project entitled “Developing standards of blood pressure in children and adolescents in Poland, OLAF” were used (20). Based on the BMI percentile values, underweight (<5th percentile), normal weight (between 5th and 85th percentile), overweight (BMI ≥85th percentile and < 95th percentile), or obesity (≥95th percentile) were determined. BMI classification for mothers’ was carried out according to the WHO guidelines: underweight (<18.5kg/m^2^), normal weight (between 18.5-24.99kg/m^2^), overweight (between 25-29.99kg/m^2^), and obesity (≥30kg/m^2^) (18).

### Statistical analysis

The statistical analysis was performed using the Statistica v.12.0 Software (StatSoftPolska Sp. z o.o., Kraków, Poland). Differences between groups were analyzed using χ^2^ test or McNemar’s test where appropriate, with Yates correction applied. For associations between taste preferences and body mass categories odds ratios (OR) with 95% confidence interval (CI) were calculated. A *P* value <0.05 was considered statistically significant.

## Results

### Perception for fat and sweet taste among children

Test results provide information on the level of taste perception for fat and sweet taste (**Table 1**). The differences in fat content in puddings were imperceptible for nearly 30% of the subjects. Another approx. 30% have correctly assessed the differences in the levels of fat taste. The remaining individuals, i.e. about 40%, had some problems with the exact ranking of fat content in the pudding samples tested, which is why they can be included in the group with average sensitivity to fat taste. The important information is that the assessments were not associated with the BMI of the tested children.

**Table 1:** Accuracy of children’s taste perception according to body mass category.

Accuracy of fat/sweet taste ratings	Body mass classification **			P value
	normal	overweight + obese	all	
high (fat)	23 (30,7%)	21 (28,0%)	44 (29,3%)	*p* = 0,6573
average (fat)	34 (45,3%)	31 (41,3%)	65 (43,3%)	
low (fat)	18 (24,0%)	23 (30,7%)	41 (27,3%)	
high (sweet)	48 (64,0%)	45 (60,0%)	93 (62,0%)	*p* = 0,7976
average (sweet)	22 (29,3%)	23 (30,7%)	45 (30,0%)	
low (sweet)	5 (6,7%)	7 (9,3%)	12 (8,0%)	

** data are expressed as n (%).

### Mothers’ taste perception vs. children’ taste perception

Although the accuracy of recognizing the intensity of fat and sweet taste was roughly similar in both groups, the disconcordance between the groups reached the statistical significance level, which was more pronounced with regards to sweet taste (**Table 2**).

**Table 2:** Accuracy of recognizing the intensity of fat and sweet taste by mothers and children, and concordance between them.

Mothers		Fat			Sweet		
Children		low	average	high	low	average	high
**Fat**	low	17 (33,3%)	20 (29,4%)	7 (22,6%)	–	–	–
	average	28 (54,9%)	25 (36,8%)	12 (38,7%)	–	–	–
	high	6 (11,8%)	23 (33,8%)	12 (38,7%)	–	–	–
**Sweet**	low	–	–	–	68 (68,7%)	23 (50,0%)	2 (40,0%)
	average	–	–	–	26 (26,3%)	18 (39,1%)	1 (20,0%)
	high	–	–	–	5 (5,1%)	5 (10,9%)	2 (40,0%)
P value for concordance		p = 0,0371*			p = 0,0171*		

** data are expressed as n (%).,* statistically significant.

### Children’s taste preferences

Taste preferences are significantly different for children with normal and excessive body weight (**Table 3**).The individuals with normal body weight choose a low fat and low sweet taste relatively more frequently than those with an overweightand obesity. High sweet compared to low sweet taste preference was associated with significantly higher probability of development of excess body weight, OR 3.77 (1.66-8.55), P=0.002. In case of high and low fat taste preference, this relationship appeared to be borderline insignificant, OR 2.42 (1.03-5.69), P=0.067.

**Table 3:** Relationship between taste preferences and body weight category among children.

Taste Preference Test	Body mass classification**		P value
	normal	overweight + obese	
low (fat)	23 (30,7%)	19 (25,3%)	*p* = 0,0173*
average (fat)	36 (48,0%)	24 (32,0%)	
high (fat)	16 (21,3%)	32 (42,7%)	
low (sweet)	37 (49,3%)	19 (25,3%)	*p* = 0,0046*
average (sweet)	22 (29,3%)	25 (33,3%)	
high (sweet)	16 (21,3%)	31 (41,3%)	

** data are expressed as n (%).,*statistically significant.

### Taste preferences of children and mothers – comparison

There were no significant differences in fat content preferences between mothers and their children, while children significantly more frequently preferred sweet or high sweet taste compared to mothers, OR 2.26 (1.42-3.58), P<0.001 (**Table 4**).

**Table 4:** Fat and sweet taste preferences for children and mother’s – comparison.

	Preference test**					
	fat taste			sweet taste		
	low	average,	high	low	average,	high
Children	42 (28,0%)	60 (40,0%)	48 (32.0%)	56 (37,4%)	47 (31.3%)	47 (31.3%)
Mothers	35 (23,3%)	49 (32,7%)	66 (44.0%)	86 (57,3%)	36 (24.0%)	28 (18.7%)
P value	0.101			0.002*		

**data are expressed as n (%).,*statistically significant.

### Mothers’ taste preferences and their body mass

A sweet taste preferences were significantly different between normal-weight, overweight and obese mothers, P=0.002.Obesity was significantly associated with sweet and high sweet taste preferences compared to normal-weight mothers. These preferences were not significantly different between normal-weight and overweight mothers (**Table 5**). No similar relationship was found for the fat taste, P=0.091.

**Table 5:** Association between mother’s sweet taste preferences and their body mass index category.

BMI classification mother (M)	Sweet taste preference									
	low sweet		sweet				high sweet			
	*N*	%	*N*	%	*OR*	*p*	*N*	%	*OR*	*p*
Normal weight	52	64.2	17	21.0	Ref.	–	12	14.8	Ref.	–
Overweight	29	63.0	7	15.2	0.74 ((0.27-1.99)	0.721	10	21.7	1.49 (0.58-3.88)	0.562
Obese (M)	5	21.7	12	52.2	7.34 (2.26-23.85)*	<0.001	6	26.1	5.20 (1.36-19.91)*	0.019*

OR, odds ratio; N, quantity; * statistically significant.

### Parental taste preferences and the incidence of overweight and obesity among children

**Figure 1:**
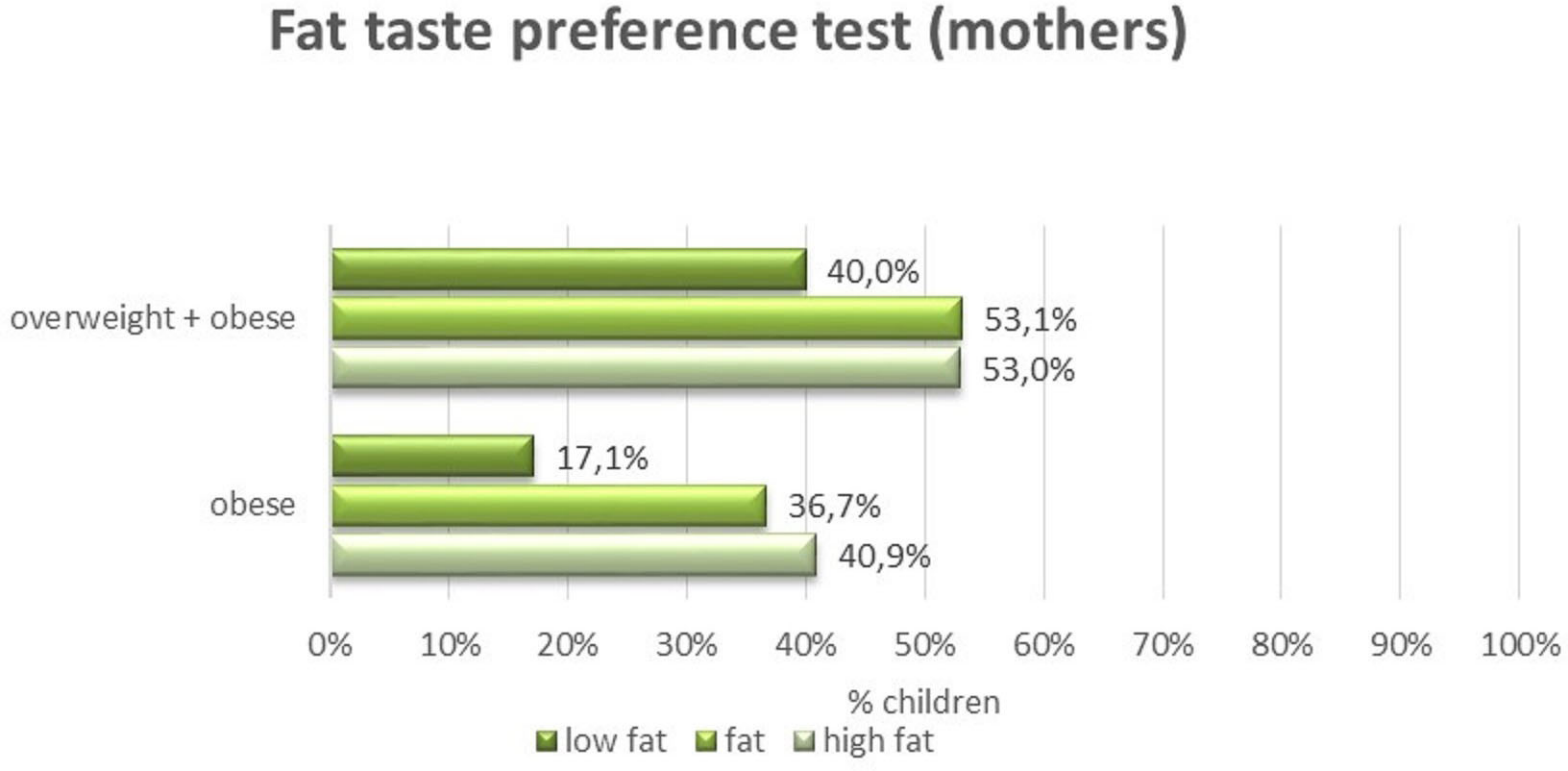
Preferences of the fat taste among mothers and the occurrence of overweight and obesity among children.

Sensitivity to fat taste among mothers is not associated with the incidence of weight disorders among children, while fat taste preferences are of great significance - the fatter the taste preferred by mothers, the greater the percentage of children with obesity (17% in case of choosing low-fat taste, and 37% and 41% in case of fat or high fat) (**Figure 1**). No similar relationship was found for the sweet taste.

## Discussion

Sensory sensations have a significant impact on people’s nutritional behavior (21). In everyday contact with food, the most pleasant is such stimulus intensity to which the consumer is accustomed. Sensitivity to each of the flavors varies widely from person to person (22). Some studies confirm that people who are sensory insensitive prefer higher concentrations of specific tastes (sour, bitter, sweet, salty, umami) (23, 24). The consequence of individual differences in the perception of tastes may be disproportions in the consumption of some dietary ingredients, especially sugar or fat, which, as a result, in those who are less sensory sensitive may lead to an increase in caloric content of the diet.The diet of people who liked to consume more sweet products was characterized by a higher energy supply and a higher consumption of carbohydrates (starch, fructose, glucose, total sugars) (25). Lim et al. suggests that the tendency towards higher sweet consumption may be due to lower sensory sensitivity (26). Most of reliable studies also point out the positive relationship between acceptance of high fat content and BMI or body weight (27–29).At the same time, obese people may be less sensitive to some unpleasant sensory fatty acids, so their appetite in some products or fatty foods is not diminished (30).Most of the available publications check the level of sensory sensitivity by comparing thresholds for individual flavors on standardized aqueous solutions. It is worth noting, however, that taste sensitivity is not always associated with preferences for stimuli at the threshold level or suprathreshold level so the lack of correlation with body weight is not necessarily surprising (31, 32). The study of such factors as the intensity of taste, hedonic sensation (“liking”), and not the threshold of sensation, are more representative for sensory measurements, because people are in contact with food every day in which flavors occur at suprathreshold levels (33).

During the experiment it was evident that the evaluators had a significant problem with the correct assessment of the fat content of various puddings. The obtained results (**Table 1**) confirmed this fact, because over 70% of the respondents rated the intensity of the fat taste incorrectly, while in the case of sweet taste as much as 62% of the respondents had no problem with the proper ranking of the intensity. Low sensitivity to fat taste is confirmed by some authors, who suggested that fat taste is not well perceived by humans and this may be a problem in sensory perception measurements (34). Despite the use of food samples instead of standardized aqueous solutions, the study did not confirm differences in the taste perception of children with proper body weight and obese children. There were also no significant differences in the perception of fat taste between parents and their children.

However, the study confirmed strong preferences for sweet taste in children. Sweet or very sweet puddings were more often chosen by children and youth (63%) than their mothers (43%) (**Table 4**) Mennella et al. (35), who also used puddings in her research presents the same conclusions regarding sweet taste preferences in children aged 5-10. Children preferred a higher concentration of sugar in both puddings and aqueous solutions. According to other reports, children are predisposed to prefer foods rich in energy, sugar and salt (36, 37).There was also a significant difference in the ability to rank samples relative to the intensity of sweet taste. Children more often did it incorrectly, as in our study, where the difference between children and mothers in this aspect was not so significant.It might be due to the higher average age of children involved in the experiment compared to Mennella’s study. In the same study it was observed that children were less willing than parents to choose puddings with a higher fat content. In our study it was noted as some literature sources suggested that preferences for sweet/fat taste were associated with overweight and obesity of children (**Table 3**) (14). Also in the case of mothers, similarly to the study by Ettinger et al. (38), preferences for sweet taste were associated with overweight and obesity, and particularly clearly with obesity (**Table 5**). Undoubtedly, many facts prove that taste preferences are related to sensory perception, food consumption and people’s body weight. However, it is not obvious how the mechanism works, which causes people with different sensitivity to flavors, different taste preferences react differently to consumed food. Ambiguous results regarding the impact of taste sensitivity on consumer choices of children withdo not help to draw objective conclusions. Sensitivity to fat taste identified as NEFA (non-esterified fatty acids) was the subject of a meta-analysis by Tucker et al. (15). It showed no differences between lean and obese people in threshold sensitivity and in assessing the suprathreshold intensity. Other such studies using NEFA provide different results. If a relationship is already detected, it is usually negative, i.e. as the body mass increases, sensitivity to NEFA decreases (39, 40). Available literature also gives examples of fat sensitivity tests using oleic acid (41), or linoleic acid as in Marinez - Ruiz et al. study (42) which confirms the inverse correlation between fat perception and BMI of the participants. Alternatively, it has been suggested that stimuli of considerable intensity, especially if they are exposed for a long time, can cause changes consisting in a permanent increase in the sensitivity threshold. Changes in stimulus sensitivity may be the greater, the longer the exposure time and the higher the stimulus concentration. A high-fat diet can lead to habituation and the need to increase stimulus levels to the desired quality in the mouth, leading to an increase in dietary fat intake and weight gain (29, 43). In Di Patrizio studies, both slim and obese people on a 4-week low-fat diet showed a higher taste sensitivity to fatty acids compared to the state at the beginning of the experiment (44). In the case of using a high-fat diet, the level of sensitivity in overweight and obese people has not changed, which may indicate that the subjects easily adapted to the permanent high-fat diet.

Parents, through their eating habits as well as everyday decisions, influence the child’s environment and influence the shaping of their sensory preferences (14, 45). The role of early nutritional experience, breastfeeding, early exposure to a wide range of products varied in taste, determine later preferences and dietary habits (46, 47). The phase of introducing a complementary feeding is the most important period of learning the taste preferences and the control of appetite in human life. Infants discover sensory impressions (texture, taste, smell) and nutritional properties (energy density) of food that is part of the diet of adults (48). From this perspective, comparing the results regarding the perception and taste preferences of children and parents is very interesting. However, in our study, comparing the results of taste preferences of children and mothers did not show significant differences. It is obvious that the newborn’s “primary” taste preferences are significantly modified in childhood by various environmental factors and may change over time as the child develops (49). Parents’ taste preferences are the result of their extensive nutritional experience related to their age and are determined more strongly by such factors as age, gender, health status, level of education or income (50). The obtained results showed the dependence of preferences regarding the taste of fat in mothers andBMI of children. This relationship may be explained by the influence of preferences - the parents’ preference for high fat content in the type and method of preparing meals prepared for the whole family (including children). Jilani et al. showed that children’s sensitivity to sweet and salty tastes is related to weight status (51). The comparison of the results of the taste sensitivity (accuracy of recognizing the intensity of taste) of children and their mothers indicates that the level of taste perception of children is correlated with the level of taste perception of mothers. It can therefore be argued that taste perception (level of taste sensitivity) can be genetically determined to a large extent. It has been established that the taste sensitivity associated with a particular gene variant has an impact on the feeling of satisfaction with food and certain eating behaviors (52, 53). It would be valuable for the results of the study to include also the fathers of participants. Our results were also limited by the small number of examined children. It should be noted that other factors like socio-cognitive determinants (e.g., parental diet, the availability of healthy food at home, income) were not included in the study. Apart from these limitations our study has several strengths. The use of a daily product (pudding) with different sugar and fat content is a strong point of the study. The protocol for testing preferences and taste perception was simple and understandable for both children and mothers, which also limited potential errors.

Proper eating patterns are especially important as there are more and more arguments indicating that impaired fat taste perception/sensitivity in obesity may result from excessive activation of the brain’s reward system, leading to an increase in the consumption of foods rich in lipids, carbohydrates and energy, and consequently overweight and obesity (54).

## Conclusions

In this study, an attempt was made to compare the preferences and taste sensitivity of children and their mothers. Puddings with different sugar and fat content were used in the research. The obtained results showed that children of mothers who preferred the more fatty products (puddings) have a higher BMI than other children. Moreover, children preferred a higher concentration of sugar in the pudding compared to theirs mothers. The result also showed that there were no significant differences in the taste perception of fat between parents and their children, although parents preferred fatty foods. However, there was no correlation between sensitivity to sweet and fat tastes and BMI of children. Our results confirm that mothers, through their eating behavior, can influence the diet of their children and ultimately their BMI. Children with normal body weight chose a low fat and low sweet taste relatively more frequently than those with an overweight. The role of parents in shaping taste preferences is very important and should be based on limiting the consumption of products rich in sugar or fat.

## Data availability statement

The raw data supporting the conclusions of this article are available on reasonable request.

## Ethics statement

The studies involving human participants were reviewed and approved by Institutional Bioethics Committee at the University of Rzeszow on 02.06.2015 (Resolution No. 15/06/2015) and by all appropriate administrative bodies. Written informed consent to participate in this study was provided by the participants’ legal guardian/next of kin.

## Author contributions

GS, MD: study design. GS: data collection. GS, MD: data analysis and interpretation. GS, MD: manuscript preparation and critical revision.

## Conflict of interest

The authors declare that the research was conducted in the absence of any commercial or financial relationships that could be construed as a potential conflict of interest.

## Publisher’s note

All claims expressed in this article are solely those of the authors and do not necessarily represent those of their affiliated organizations, or those of the publisher, the editors and the reviewers. Any product that may be evaluated in this article, or claim that may be made by its manufacturer, is not guaranteed or endorsed by the publisher.
